# Data-Driven Design of Nickel-Free Superelastic Titanium Alloys

**DOI:** 10.3390/ma17081793

**Published:** 2024-04-13

**Authors:** Haodong Chen, Wenjun Ye, Songxiao Hui, Yang Yu

**Affiliations:** 1 State Key Laboratory of Nonferrous Metals and Processes, China GRINM Group Co., Ltd., Beijing 100088, China; 2 GRIMAT Engineering Institute Co., Ltd., Beijing 101407, China; 3 General Research Institute for Nonferrous Metals, Beijing 100088, China; 4 GRINM (Guangdong) Institute for Advanced Materials and Technology, Foshan 528051, China

**Keywords:** nickel-free superelastic titanium alloys, superelastic strain, machine learning, Bayesian optimization

## Abstract

In this paper, a CatBoost model for predicting superelastic strains of alloys was established by utilizing features construction and selection as well as model filtering and evaluation based on 125 existing data points of superelastic titanium alloys. The alloy compositions of a TiNbMoZrSnTa system were optimized and three nickel-free titanium alloys with potentially excellent superelastic properties were designed using the Bayesian optimization algorithm using a superelastic strain as the optimization target. The experimental results indicated that only Ti-12Nb-18Zr-2Sn and Ti-12Nb-16Zr-3Sn exhibited clear superelasticity due to the absence of relevant information about the alloys’ β stability in the machine learning model. Through experimental optimization of the heat treatment regimens, Ti-12Nb-18Zr-2Sn and Ti-12Nb-16Zr-3Sn ultimately achieved recovery strains of 4.65% after being heat treated at 853 K for 10 min and 3.01% after being heat treated at 1073 K for 30 min, respectively. The CatBoost model in this paper possessed a certain ability to design nickel-free superelastic titanium alloys but it was still necessary to combine it with existing knowledge of material theory for effective utilization.

## 1. Introduction

NiTi alloys, due to their unique comprehensive mechanical properties such as high corrosion resistance [1], excellent biocompatibility [2], shape memory effect and superelasticity [3], provide a perfect solution to the challenges faced in minimally invasive surgical procedures [4]. The maximum recovery strain (${ɛ}_{r}^{\max}$) of a NiTi alloy can reach 8%, generally higher than that of nickel-free superelastic titanium alloys [3]. However, Ni is a toxic, allergenic and mutagenic element for humans, posing significant safety risks when used within the human body [5]. The design of nickel-free superelastic titanium alloys with excellent superelasticity is an urgent problem that needs to be addressed.

Nickel-free superelastic titanium alloys rely on the reversible transformation of the metastable β phase to the orthorhombic martensite phase (α″) under stress for their superelasticity. High levels of β stability lead to a martensitic transformation initiation stress (σ_SIM_) that is higher than the yield strength of the β phase, hindering the reversibility of the martensitic transformation and impeding the manifestation of superelasticity. A lower β stability will result in the generation of a significant amount of α″ phase during water quenching, reducing the overall recovery strain (ε_r_). The influence of alloy elements on the stability of the β phase is significant, making the manifestation of superelasticity in titanium alloys sensitive to alloy compositions [6]. The elements Nb, Mo, Zr, Sn and Ta can, to varying degrees, reduce the stability of the β phase in titanium alloys. Each 1 at.% Nb can reduce the martensitic start temperature (M_s_) of Ti-Nb binary alloys by 40 K [7,8,9], Ti-Nb-Zr alloys by 40–70 K [10] and Ti-Nb-Mo alloys by 25–30 K [11]. Each 1 at.% Zr can reduce the M_s_ of Ti-Nb-Zr alloys by about 40 K [12] and Ti-Mo-Zr-Sn alloys by 38 K [13]. Each 1 at.% Sn can reduce the M_s_ of Ti-Mo-Zr-Sn alloys by 150 K [13]. Each 1 at.% Ta can reduce the M_s_ of Ti-Ta and Ti-Nb-Ta alloys by 30 K [8,14]. Different β-stabilizing elements reduce the M_s_ of titanium alloys to varying degrees. Therefore, it is necessary to rationally combine alloy elements to ensure that the M_s_ of the alloy is near room temperature to achieve superelasticity.

Thermo-mechanical processing significantly influences the ultimate superelastic performance of superelastic titanium alloys. Li et al. [15] conducted aging treatment on the Ti-40Zr-8Nb-2Sn alloy at 573 K for 1 h. The addition of a high content of Zr hindered the growth of the isothermal ω phase (ω_iso_), resulting in the precipitation of nano-sized ω_iso_ in the alloy. The nano-sized ω_iso_ enabled the alloy to achieve an ideal balance of high yield strength and high ductility with a large superelastic strain (ε_SE_) of 5%. Sun et al. [16]. conducted an extremely short-term aging heat treatment at 873 K for 6 min on the Ti-20Nb-6Zr alloy to obtain ultrafine recrystallized β grains with sizes ranging from 1 to 2 μm. This treatment effectively improved the alloy’s mechanical properties, resulting in a recoverable strain (ε_r_) of 3.2% and a tensile strength of 750 MPa. Fu et al. subjected the Ti-18Zr-4.5Nb-3Sn-2Mo alloy to heat treatment at 1173 K for 5 min, obtaining an ɛ_r_ of 6.2% which was attributed to fine-grain strengthening and the well-developed {001}_β_<110>_β_ texture. For a specific alloy, it is crucial to carefully choose the appropriate thermo-mechanical processing scheme to optimize its superelastic performance.

The superelasticity of titanium alloys is influenced by various factors and traditional alloy design methods are no longer sufficient to meet the requirements of rapid advancements in new alloys design today. Machine learning methods demonstrate enormous potential for analyzing material knowledge from data as they can greatly reduce the cost, risk and time associated with material research and development [17]. The most common application of machine learning is to establish the relationship between existing data features and material properties and then extend this relationship to new systems to predict the performance of new data points [18]. The transformation temperatures, superelasticity and thermal hysteresis phenomena of NiTi alloys have been widely investigated using machine learning techniques, while there is limited research on machine learning for nickel-free superelastic titanium alloys [19]. Xue et al. proposed a method for predicting transition temperatures using three features or material descriptors related to chemical bonds and atomic radii of elements in NiTi alloys [20]. Furthermore, the thermal hysteresis of NiTi alloys was predicted and a Ti_50.0_Ni_46.7_Cu_0.8_Fe_2.3_Pd_0.2_ alloy with the minimum thermal hysteresis was designed using a global optimization approach [21]. Wang et al. utilized data from traditional Ti alloys and Ti-rich quaternary high-entropy alloys as a basis to predict the phase composition of the alloy system and they designed a metastable Ti_48_Zr_20_Hf_15_Al_10_Nb_7_ high-entropy alloy with superelastic properties [22]. Although the aforementioned studies were successful in designing alloys with superelasticity, they failed to establish a direct relationship between material descriptors and the final mechanical properties. A more intuitive connection between material descriptors and superelastic properties is still must be established.

In this paper, a predictive model for the maximum superelastic strain (${ɛ}_{\mathrm{SE}}^{\max}$) was established by considering features such as alloy preparation processing and elemental characteristics based on the data for existing traditional nickel-free superelastic titanium alloys. The use of Bayesian optimization algorithms enabled the rapid design of novel titanium alloys with significant potential for superelasticity. The heat treatment regimens of each alloy were optimized to achieve the optimal superelastic performance.

## 2. Materials and Methods

### 2.1. Dataset Establishment

All data in the nickel-free superelastic titanium alloy dataset were collected from the TiNbMoZrSnTa alloy system, which were often accompanied by information about their well-established shape memory effects or superelasticity gained by consulting references [6]. Each data point contained the alloy composition as well as the processing (cold rolling reduction) and heat treatment (solution temperature and time) parameters that significantly affect the alloy’s properties. The target performance was ${ɛ}_{\mathrm{SE}}^{\max}$.

All alloys were obtained through arc melting, cold rolling and annealing to ensure data consistency and ${ɛ}_{\mathrm{SE}}^{\max}$ was all obtained by cyclic loading-unloading experiments. In addition, there was some variation in different studies for the same alloys, so only the most trustworthy data point was utilized. Furthermore, outliers were also rejected during data preprocessing.

**Figure 1:**
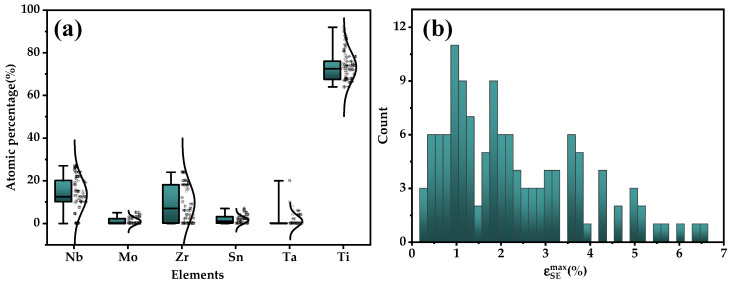
Distribution of (**a**) alloy compositions and (**b**) ${ɛ}_{\mathrm{SE}}^{\max}$ in dataset.

After filtering, 125 data points were finally collected as the nickel-free superelastic titanium alloy dataset. The database contained different alloy compositions and different processes for certain compositions. The distribution of alloy compositions and superelastic strain values in the database are shown in Figure 1a,b, respectively. Naturally, alloys of the Ti-Nb-Zr system dominate the dataset and the range of superelastic strain was 0.26–6.48. Figure 1b shows that the ${ɛ}_{\mathrm{SE}}^{\max}$ of nickel-free titanium alloys were mainly concentrated in the range of 0~4%, with few alloys exceeding 4%.

### 2.2. Feature Construction

The superelasticity of titanium alloys was influenced by a number of factors such as the addition of alloying and interstitial elements, as well as heat treatment and deformation parameters [23,24]. The atomic percentages of alloys were taken for granted as features. For the different heat treatment regimes, only the most widely used cold rolling and annealing process was selected. Ultimately, the features selected in the material preparation and processing were cold rolling reduction, annealing time and temperature.

Specifically, the superelasticity of titanium alloys arises from stress-induced martensitic phase transformations. Therefore, only metastable β titanium alloys can exhibit superelasticity at room temperature. Designing titanium alloys with room-temperature superelasticity imposes requirements on the stability of the β phase [6]. Several traditional alloy design methods involving the molybdenum equivalent method, d-electron concept and electron/atom (e/a) ratio all have proposed parameters related to the stability of the β phase in titanium alloys [25,26]. Consequently, the molybdenum equivalent (Mo), two parameters of d-electron concept (Bo and Md) and the average valence electron concentration (VEC) were added to the feature set.

In addition, a set of element features (e.g., relative atomic mass, number of periods and groups in the periodic table of elements) and physical (e.g., Young’s modulus, melting point and thermal expansion coefficient) and chemical (e.g., Pauling electronegativity) parameters were selected as features. For maximizing the search for features correlated with superelastic strain, the initial feature set extensively encompassed various features related to thermodynamics, electricity, physics, chemistry and other factors possibly associated with alloy performance. Such features are listed in Table 1. In order to extract potential information from the above-mentioned features, the linear mixture rule(x_1_), the reciprocal mixture rule (x_2_), the deviation rule(x_3_) and the discrepancy(x_4_) [27,28,29] were calculated based on the atomic percentage compositions and added to the feature set. The four types of features can be calculated as follows:

(1) $$x_{1}=\sum_{i}^{n}a_{i}x_{i}$$

(2) $$x_{2}=({\sum_{i=1}^{n}\frac{a_{i}}{x_{i}})}^{-1}$$

(3) $$x_{3}=\sqrt{\sum_{i=1}^{n}a_{i}{\left(x_{i}-x_{1}\right)}^{2}}$$

(4) $$x_{4}=\sqrt{\sum_{i=1}^{n}a_{i}{\left(1-{x_{i}/x}_{1}\right)}^{2}}$$

where a_i_ is the atom fraction and the x_i_ is features related to the alloy compositions. The initial feature set ultimately contained a total of 87 features. 

### 2.3. Feature Selection

The significance of feature selection lies in eliminating redundant and irrelevant features which means selecting as few features as possible while retaining the important information contained in the feature set. Fewer features help to simplify the model and enhance the model’s interpretability [30,31]. Three steps of feature selection including Pearson correlation coefficient (PCC), recursive feature elimination (RFE) and best subset selection (BSS) were applied in this paper.

The first step was to use PCC to remove redundant features. The Pearson correlation coefficient can be calculated as follows:

(5) $$p_{x,y}=\frac{\sum_{i=1}^{n}\left(x_{i}-\bar{x})(y_{i}-\bar{y}\right)}{\sqrt{\sum_{i=1}^{n}{\left(x_{i}-\bar{x}\right)}^{2}}\sqrt{\sum_{i=1}^{n}{{(y}_{i}-\bar{y})}^{2}}}$$

where $x_{i}$ and $y_{i}$ are the values of two different features respectively; $\bar{x}$ and $\bar{y}$ represent the mean of $x_{i}$ and $y_{i}.p_{x,y}$ was used to measure the linear correlation between features. The range of $p_{x,y}$ was between −1 and 1. The closer the absolute value of $p_{x,y}$ was to 1, the stronger the correlation between features. Highly correlated features were considered to contain similar valuable information. In this paper, features with a $p_{x,y}$ greater than or equal to 0.95 were considered highly correlated but only one could be retained. Which feature to retain was determined by comparing the Pearson correlation coefficient between features and ${ɛ}_{\mathrm{SE}}^{\max}$. Features with a higher correlation to the superelastic strain would be retained. PCC solely examined the relationships between features and conducted an initial screening of the feature set, removing redundant features.

The second step was RFE based on the feature importance. RFE first built a machine learning model using the selected feature above and used the model’s built-in evaluation parameters to rank the importance of features. Then, the least important feature based on the ranking was removed and the above steps were repeated with the remaining features until the number of features was reduced to 1. The performance of each iteration’s model was recorded to facilitate the selection of the optimal number features.

The final step was BSS which used an exhaustive method to enumerate all feature subsets and select the best feature combination. RFE selected some important features but it could not consider all combinations. Based on the selected features in RFE, the final step involved modeling and evaluating all of the feature combinations. The best feature subset could be obtained by comparing differences in model performance.

### 2.4. Machine Learning

All machine learning processes were carried out using the programming language Python. In order to mitigate the impact of different scales among features on calculations and to meet the requirements of neural network computations, the data were normalized as follows:

(6) $$X\prime =\frac{x-\mu }{s}$$

where x and x′ are actual value and normalized value, the symbols μ and s represent the mean and standard deviation of the sample, respectively.

The CatBoostRegressor (CatBoost) machine learning algorithm (default parameters) was used for RFE. The feature importance was also an inherent parameter of the CatBoost model. After two steps of filtering features, six different models including Support Vactor Regressor (SVR), CatBoost, KNeighborsRegressor (KNN), Backpropagation Neural Network (BPNN), Gaussian process Regressor (GPR) and XGBRegressor (XGBoost) were applied to select a the most suitable for the prediction of ${ɛ}_{\mathrm{SE}}^{\max}$. The performances of the models were evaluated using the coefficient of determination (R^2^) and mean absolute error (MAE) shown below:

(7) $$\mathrm{MAE}=\frac{1}{n}\sum_{i=1}^{n}\left|y_{i}-\hat{y_{i}}\right|$$

(8) $$R^{2}=1-\frac{\sum_{i=1}^{n}{\left(y_{i}-\hat{y_{i}}\right)}^{2}}{\sum_{i=1}^{n}{\left(y_{i}-\overset{-}{y}\right)}^{2}}$$

where n is the number of samples in the dataset, $\bar{y}$ is the mean of all experimental values in the dataset and yᵢ and $\hat{y_{i}}$ are the experimental values and the corresponding predicted values by the machine learning model for each sample in the dataset, respectively. Five-fold cross-validation (5-fold cv) was employed during the feature selection and model evaluation processes for model establishment and assessment to avoid randomness caused by a small dataset.

### 2.5. Experimental Validation

**Figure 2:**
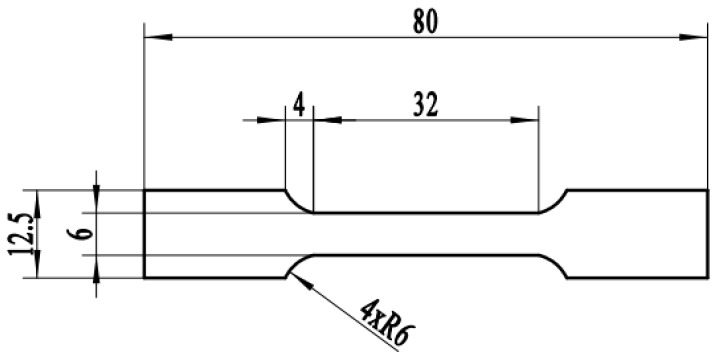
Tensile specimen dimension schematic.

First, 10 kg alloy ingots were melted through the VISM-50 cold crucible levitation melting equipment using high-purity Ti (99.99%), Nb (99.99%), Zr (99.99%) and Sn (99.99%). The ingots were subjected to three forging processes after peeling and removing the riser to obtain a slab. The slabs were hot -rolled to 16 mm after being incubated at 800 °C for 45 min. The hot-rolled sheets were coated with an anti-oxidation layer on the surface, followed by solution treatment at 950 °C for 1 h. After water quenching, the surfaces were milled to achieve a clean finish. Subsequently, the sheets underwent cold rolling with a single-pass reduction not exceeding 0.3 mm. The amount of rolling deformation was 0.8 with no intermediate annealing performed during the cold rolling processes. The specimens for superelasticity testing were all taken along the rolling direction from the cold-rolled sheets and their dimensions are shown Figure 2. Subsequently, the specimens were heat -treated at temperatures ranging from 600 to 900 °C for 10 min (AC) or 30 min (WC). The surface oxide scales were removed through acid pickling and the composition of the acid pickling solution was HF: HNO_3_: H_2_O = 1:3:7. Cyclic loading-unloading experiments were initiated by stretching the specimens to a strain of 1.5% followed by unloading to 10 N. Subsequently, the specimens were cyclically stretched and unloaded in increments of 0.5% strain until they fractured or reached 10% strain before unloading. The strain rate for both loading and unloading in the experiments was maintained at 1 × 10^−3^ s^−1^.

## 3. Results

### 3.1. Feature Selection and Model Establishment

**Figure 3:**
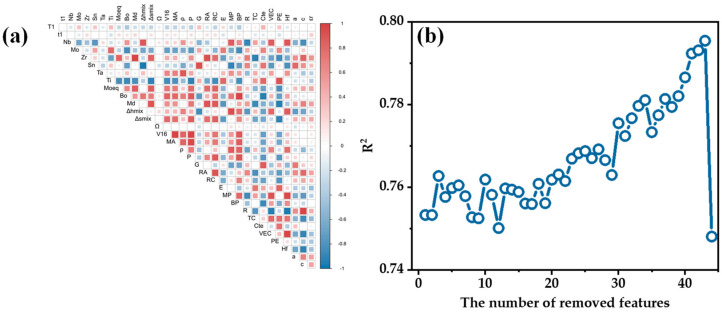
Results of the first two steps of feature selection: (**a**) heatmap of correlations for partial features; (**b**) variation in R^2^ of the models in RFE with the number of removed features.

The Pearson correlation coefficients between all of the features have been calculated. The Pearson correlation heatmap for partial features is shown in Figure 3a. The more complete the pie chart, and the darker the colors, the higher the correlation between features. PCC significantly reduced the redundancy of the feature set, reducing the number of features from 87 to 45.

On the basis of 45 features, RFE was conducted, and the performances of various models were evaluated using 5-fold cv with the R^2^ metric. The process of recursive feature elimination is shown in Figure 3b. It can be observed that the R2 of the model did not show significant changes with the progress of the iterations when removing a relatively small number of features. There was only a very slight increase, indicating that the removal of features had a minimal impact on the model’s performance at this stage. R^2^ decreased from 0.795 to 0.748 when the number of removed features increased from 42 to 43. This significant change in R^2^ indicated that the removal of the specific feature at this point was more impactful. Removing this feature caused the feature set to lose crucial information leading to a decline in predictive accuracy. In this case, the removed feature was T1 which included data from two heat treatment processes: low-temperature short-time annealing and high-temperature solution treatment. The material’s properties under these two processes exhibited a significant difference making T1 a crucial feature affecting the superelastic strain. The preparation and processing parameters of the alloys were retained taking into account the features influencing the actual alloy system. Among them, t1 was excluded in the 33rd iteration. The feature set before the removal of t1 was selected as an alternative feature set (13): t1, Zr, a, Moeq, VEC4, ρ4, Hf4, PE4, E, MP3, T1, strain, E2.

**Figure 4:**
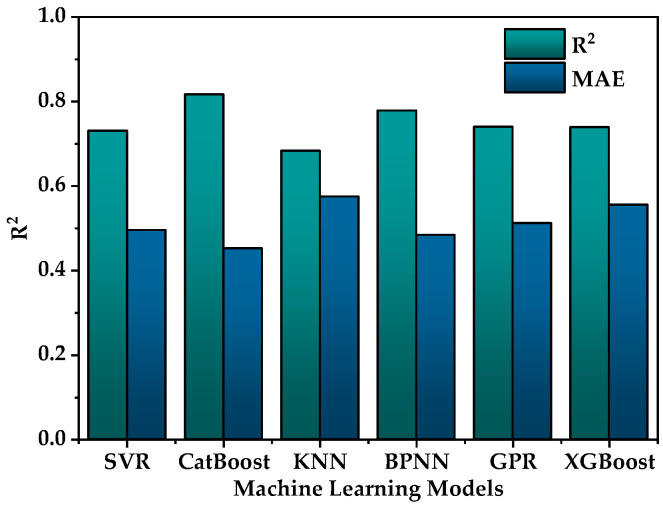
A comparison of the performances of various models.

**Figure 5:**
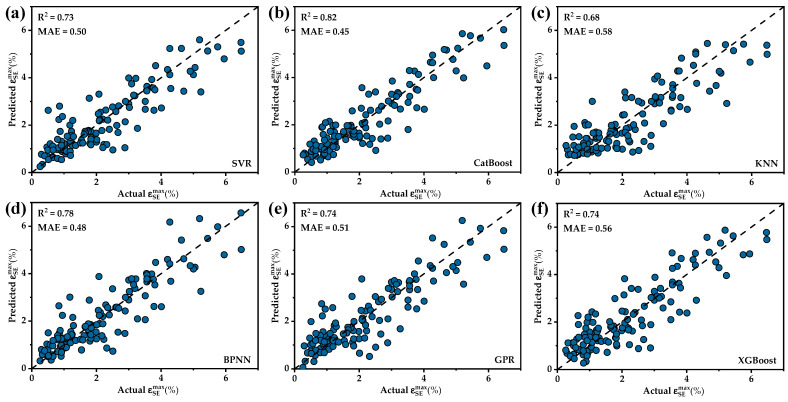
A comparison between predicted values and actual values for each model: (**a**) SVR; (**b**) CatBoost; (**c**) KNN; (**d**) BPNN; (**e**) GPR; (**f**) XGBoost.

Various machine learning models were employed based on the feature set filtered through RFE to avoid the influence of a single machine learning model on BSS as well as to select the most suitable machine learning model for the ${ɛ}_{\mathrm{SE}}^{\max}$ dataset. These models included SVR, CatBoost, KNN, BPNN, GPR and XGBoost, totaling six different machine learning models. Each model underwent hyperparameter tuning and the results of hyperparameter adjustments for each model are shown in Table 2. The model evaluation was conducted using leave-one-out cross-validation (LOOCV) and a comparison of the performances of the various models is shown in Figure 4. The CatBoost model (R^2^ = 0.82, MAE = 0.45) exhibited a higher R^2^ and a lower MAE, demonstrating a significantly better performance compared to the other five models and showcasing superior predictive capabilities. As shown in Figure 5, the comparison between predicted values and actual values for each model revealed that most models provide similar predictions for the majority of the data. However, the CatBoost model outperformed the other models, especially on a few data points where larger deviations occurred. In summary, CatBoost was chosen as the machine learning model for the next feature selection step.

**Figure 6:**
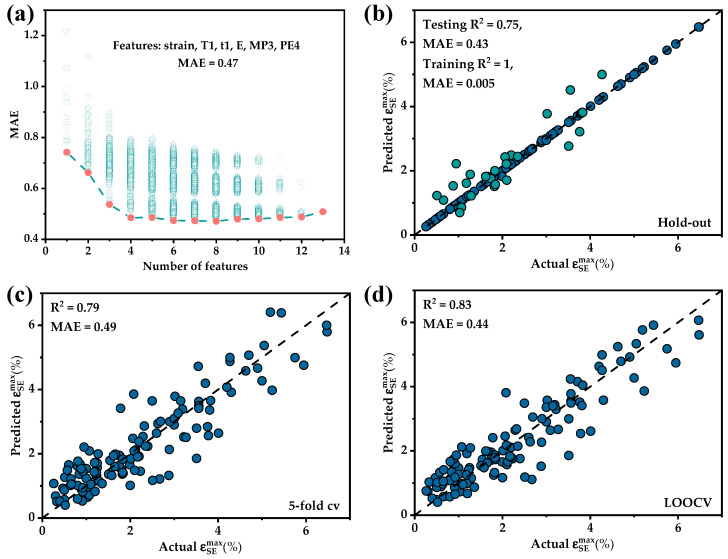
The results of BSS and the evaluation results for different dataset partitions: (**a**) variation of MAE of the models with the number of features; (**b**) hold-out; (**c**) 5-fold cv; (**d**) LOOCV.

All different combinations of the 13 features were modeled and evaluated in best feature subset selection as shown in Figure 6a. The best model was achieved with seven features but the performance improvement from six to seven features was marginal. Six features were chosen in order to select as few features as possible. The CatBoost model’s MAE was 0.51 with the feature set including strain, T1, t1, E, MP3 and PE4. The specific meanings of the six features were cold rolling reduction (strian), annealing temperature (T1), annealing time (t1), average elastic modulus (E), standard deviation of melting point (MP3) and discrepancy of Pauling electronegativity (PE4).

A CatBoost model was established based on the final feature set. Data partitioning could significantly impact the evaluation results of the model due to the limited amount of data. Therefore, three different data partitioning methods were employed for model evaluation: the holdout method (testing set = 0.2), 5-fold cv and LOOCV. As shown in Figure 6b–d, CatBoost predicted the training data almost perfectly, but biases were often observed for the test set. The magnitudes of data bias were relatively consistent among the three data partitioning methods. Therefore, it was considered that data partitioning had not significantly affected the predictive results. The data points were distributed almost symmetrically on both sides of the 45° line indicating that the predicted range was generally reasonable. However, they did not fall precisely on the line because of the vastness of the data space combined with the insufficient and uneven distribution of data points in the dataset. Additionally, variations in alloy preparation and processing, data collection processes and other factors across different studies could have affected the accuracy of the data. The proportion of small superelastic strain data was relatively large and predictions for such data tended to be closer to the true values. Conversely, the prediction error significantly increased when the superelastic strain exceeded 2.5%. Therefore, the performance of this model might be less satisfactory when predicting data with a high superelastic strain.

### 3.2. New Alloy Design

After successfully establishing the correlation between key features and superelastic strain, optimizing the alloy composition for excellent performance held significant engineering significance. Although exhaustive optimization using the established machine learning algorithm was accurate, the significant amount of time it consumed was not acceptable. A Bayesian optimization algorithm was employed to optimize the alloy compositions with excellent performance based on the Optuna framework in this paper. The Bayesian optimization analyzed the existing data points as a prior distribution. Subsequently, it used an acquisition function to analyze the prior distribution and selected the location with the highest probability of extreme values for testing. The test results were then added to the prior distribution to form the posterior distribution which serves as the prior distribution for the next iteration. This process was repeated until the allotted time or the maximum number of iterations was reached. The optimization problem in this paper can be described as follows:

Maximize F (Nb, Mo, Zr, Sn, Ta)

Subject to

- 0 ≤ Nb ≤ 25 at.%,
- 0 ≤ Mo ≤ 5 at.%,
- 0 ≤ Zr ≤ 30 at.%,
- 0 ≤ Sn ≤ 5 at.%,
- 0 ≤ Ta ≤ 5 at.%,
- Nb + Mo + Zr + Sn + Ta + Ti = 100 at.%,
- Zr < Ti,
- strain = 0.985
- T1 = 1173 K,
- t1 = 30 min.

Where F is the final CatBoost model. Iterative optimization could commence after the optimization direction and range had been determined. To seek nickel-free titanium alloys with superior superelastic performance, the optimization direction was maximization. The content ranges of each alloy element were initially determined based on that of the dataset. The process parameters (strain, T1, t1) were chosen to be the most commonly used parameters in the database.

**Figure 7:**
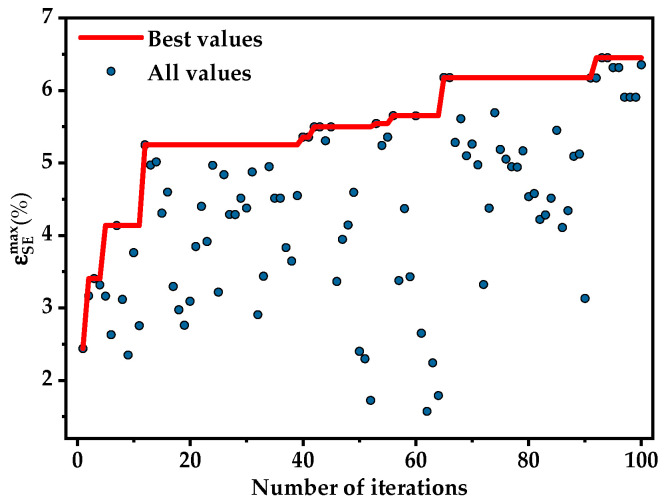
The evolution of the best value in Optuna optimization.

The variation in the best ${ɛ}_{\mathrm{SE}}^{\max}$ during the iterative process with the number of iterations was shown in Figure 7. A total of 100 iterations were conducted and the ${ɛ}_{\mathrm{SE}}^{\max}$ exceeded 6% within just 65 iterations. The optimal value remained unchanged after 90 iterations at which point the optimization process might have entered a local optimum. The optimization process was repeated multiple times to prevent the optimization process from becoming stuck in a local optimum.

It is worth noting that the optimization problem in this article involved the challenge of seeking multi-dimensional solutions for a single objective which might lead to the generation of unreasonable solutions which would require the application of a prior knowledge of materials to assess whether the optimized alloy compositions met the requirements. Most importantly, the final feature set in this study did not include features related to β stability. It can be argued that this feature set could not accurately assess the stability of the alloy. It can be anticipated that the M_s_ of some designed alloys were not in the vicinity of room temperature and they might not even be metastable β titanium alloys, and thus would be unable to exhibit superelastic performance. The establishment of an M_s_ prediction model has been attempted before. However, it was difficult to accurately measure through differential scanning calorimetry (DSC) tests due to the small enthalpy change in the martensitic transformation of titanium alloys. The determination of the M_s_ of metastable titanium alloys could only be achieved through tensile testing at different temperatures leading to limited data being available. In summary, the estimation of β stability and the rationality of alloy composition in this article could only be determined through manual judgment. Finally, three alloy compositions were selected for experimental validation, as shown in Table 3. Among them, Alloy Ⅰ was the alloy with the optimal ${ɛ}_{\mathrm{SE}}^{\max}$ obtained during the optimization process. Alloys Ⅱ, mentioned in Li’s paper [32], and Ⅲ were selected for validation to assess whether the effect of 1 at.% Sn in increasing β-phase stability could be replaced by 2 at.% Zr. Additionally, the predicted performance of Alloys Ⅱ and Ⅲ were superior to 4%.

### 3.3. Feature Analysis

**Figure 8:**
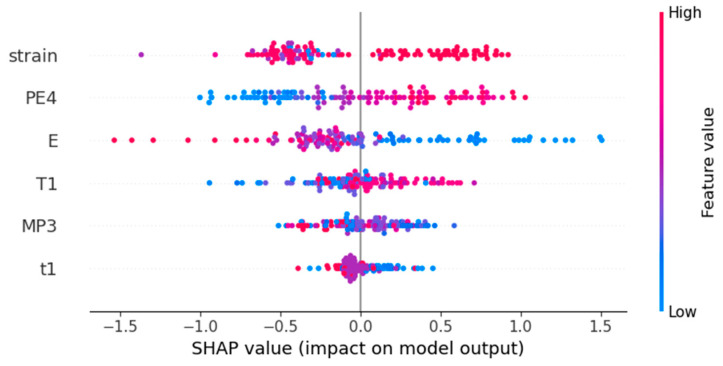
Global feature explanation chart.

Machine learning possessed high predictive capabilities. However, black-box models lacked explanations for the models’ decisions. The Shapley Additive explanations (SHAP) method was employed to analyze the influence of each feature on the superelastic strain variable in the optimal CatBoost model. SHAP is a novel machine learning model interpretation method based on game theory, which can calculate the marginal contributions (SHAP values) of features to the model’s output, and provide explanations for black-box models at both global and local levels. Figure 8 illustrated the global interpretation of the feature set, depicting the impact of each feature value on ${ɛ}_{\mathrm{SE}}^{\max}$. The *x*-axis represented the SHAP values corresponding to feature values, indicating the degree and direction of the features’ influence on ${ɛ}_{\mathrm{SE}}^{\max}$. Positive values signified an increase in ${ɛ}_{\mathrm{SE}}^{\max}$, while negative values indicate a decrease. The *y*-axis represented feature values with colors representing their magnitude (the red for higher values and blue for lower values). Additionally, the *y*-axis was sorted by feature importance with decreasing importance from top to bottom. It can be observed that a larger range of SHAP values corresponds to higher feature importance. Features with higher importance, such as PE4, E and T1, demonstrated a more significant influence on the ${ɛ}_{\mathrm{SE}}^{\max}$ variable as their values changed.

**Figure 9:**
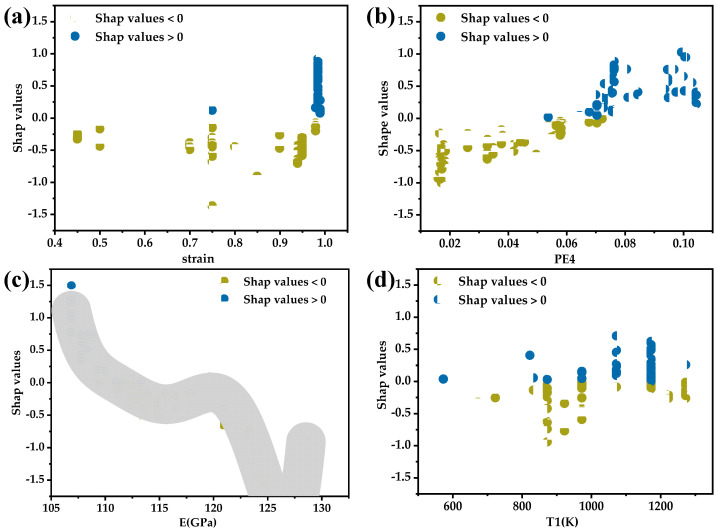
Feature dependency figures of (**a**) strain; (**b**) PE4; (**c**) E; (**d**) T1.

Feature dependency figures were plotted in Figure 9 to observe the impact of individual features on the predicted values more precisely. The patterns of feature value changes of MP3 and t1 did not show a clear influence on superelasticity, so no analysis was conducted. In Figure 9a,d, the respective impacts of feature strain and T1 on the model predictions have been demonstrated. Both features were material preparation and processing features and the data for these features were relatively discrete. In Figure 9a, the closer the cold-rolling deformation was to 1, the more positive its impact on the superelastic strain. A higher cold-rolling deformation induced high-density dislocations in the titanium sheets, promoting nucleation of the α and ω phases during subsequent annealing, resulting in a higher yield strength and an enhanced superelastic strain [15,33]. From a practical perspective, cold rolling of thick titanium alloy plates is challenging, typically commencing from a thickness of 10 mm. A large deformation exceeding 95% results in the final sheet product having a sheet thickness ranging from approximately 0.1 mm to 0.5 mm. The extremely small grain size and sheet thickness in cold-rolled sheets prevent the grains from becoming excessively large after annealing.

In Figure 9d, it was observed that the different heat treatment temperatures almost all contained data points adversely affecting the predicted values. The predominantly positive impact of annealing at 1173 K on superelasticity was due to the higher cold rolling rates in the database which required annealing above the β phase transformation point followed by water quenching to retain the entire β phase and induce recrystallization. It could be predicted that, for samples with lower cold-rolling deformation, annealing at higher temperatures may result in an increase in grain size, consequently reducing the material’s strength. In this scenario, a reasonable mid-to-low-temperature short-term annealing could control the material’s grain size, retain a certain dislocation density, enhance the alloy’s yield strength and, in turn, improve the superelastic properties of the alloy. Superelasticity occurs only when the yield strength of the alloy is greater than the onset stress of martensitic transformation. The impact of annealing temperature on superelasticity is complex and needs to be considered in conjunction with cold rolling deformation and the phase transformation temperatures of the alloy.

Figure 9b shows the change in SHAP values with the PE4 feature values. It can be observed that, with the increase in PE4, the influence on ${ɛ}_{\mathrm{SE}}^{\max}$ gradually tends towards being positive and approximately linear. The larger the Pauling electronegativity value, the stronger the attraction of the atom to the shared electrons. An increase in the PE4 feature value indicates a greater difference in electronegativity between alloy elements, enabling the formation of stronger bonds, thus increasing the strength of the alloy [34]. A higher critical slip stress is often more favorable for the manifestation of superelasticity. Figure 9c illustrates the influence of feature E on the superelastic strain. The SHAP values of E initially decreased with the increase in feature values, then stabilized and subsequently continued to decrease. A high elastic modulus implied high bond energy, which was closely related to the mechanical properties of materials. It can be confirmed that there was a certain regularity between E and superelastic performance. The pattern in Figure 9c is consistent with the fact that superelastic titanium alloys often have lower elastic modulus. Feature E and PE4 were both derived from the calculation of elastic modulus and Pauling electronegativity based on alloy compositions. In essence, they contained a wealth of compositional information.

### 3.4. Experimental Validation

**Figure 10:**
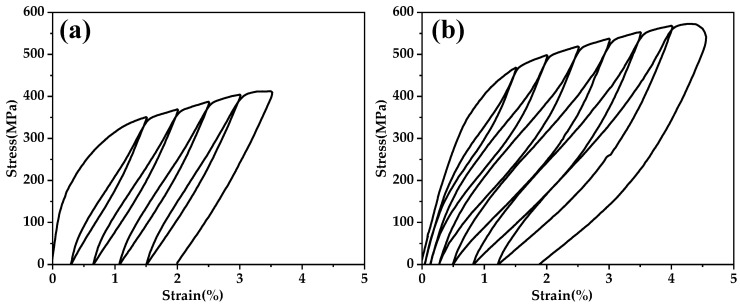
Tensile loading-unloading curves of (**a**) Alloy Ⅱ and (**b**) Alloy Ⅲ solution treated at 1173 K for 30 min.

In order to validate the feasibility of the alloy design using the machine learning model proposed in this paper, experiments were conducted to validate the three alloys designed. Table 4 presented the chemical compositions of the alloy ingots produced by the suspension melting furnace. Figure 10 shows the tensile loading-unloading curves of two alloys solutions treated at 1173 K for 30 min followed water cooling. Alloy Ⅰ did not exhibit superelasticity, confirming the earlier hypothesis that the optimization process might yield unreasonable compositions. The fundamental reason for this was that Alloy Ⅰ had an excessively high β stability, preventing stress-induced phase transformation in the β phase. This lack of the necessary conditions hindered the manifestation of superelasticity. The Ti-11Nb-24Zr-2Sn alloy had been proven to lack superelasticity at room temperature due to its excessively high β stability [35]. The addition of Zr elements tended to lower the M_s_ of titanium alloys, so the incorporation of more Zr in Alloy Ⅰ indicated that was unlikely to exhibit superelasticity at room temperature. The solid-solutions alloys Ⅱ and Ⅲ exhibited an ${ɛ}_{r}^{\max}$ of 2.78% and 1.5% in Figure 10a,b, respectively. Their ${ɛ}_{\mathrm{SE}}^{\max}$ were 1.17% and 1.88%, respectively, which evidently fell short of the well-performing outcomes predicted by machine learning. The addition of O and H elements could result in a decrease in the cold formability of titanium alloys. In this paper, the ingots, due to multiple remelting steps during the melting process, introduced O and H elements, limiting the cold deformation capability to only 80%. The ${ɛ}_{\mathrm{SE}}^{\max}$ were 1.84% and 1.99% for each predicted based on the actual cold rolling reduction. Evidently, the solution treatment at 1173 K for 30 min was not suitable for the alloy with an 80% cold rolling reduction discussed in this paper.

**Figure 11:**
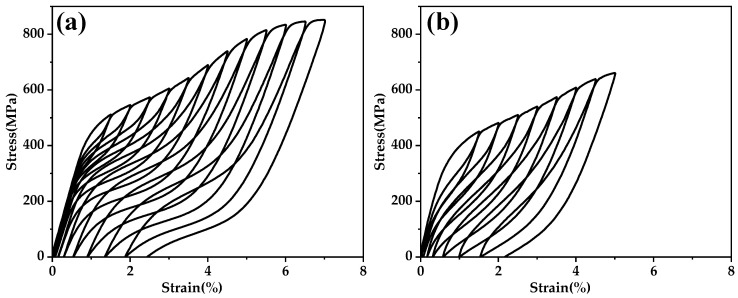
Tensile loading-unloading curves of (**a**) Alloy Ⅱ heat treated at 853 K for 10 min and (**b**) Alloy Ⅲ heat treated at 1073 K for 30 min.

The heat treatment regimens were appropriately adjusted to demonstrate the superelastic performance of the two alloys as shown in Figure 11. Alloy Ⅱ and Ⅲ achieved their optimal superelastic performance after heat treatment at 853 K for 10 min and 1073 K for 30 min, respectively. They achieved an ${ɛ}_{r}^{\max}$ of 4.65% and 3.01%, and ${ɛ}_{\mathrm{SE}}^{\max}$ of 3.19% and 2.08%, respectively. Using the optimized heat treatment regime as the input, the prediction results of the CatBoost model were 2.19% and 2.25%, respectively. In reality, the superelastic performance of Alloy Ⅱ was better than that of Alloy Ⅲ, suggesting that 1 at.% Sn cannot fully replace 2% at of Zr.

Despite the absolute prediction error being less than 1% strain, it still fell short of the desired outcome. The cold rolling and heat treatment of the alloy system were actually aimed at obtaining smaller equiaxed grains and better texture orientation to improve superelasticity. However, the data available in this paper were already very limited, making it even more challenging to obtain information about the microstructure of nickel-free superelastic titanium alloys. Therefore, the next optimization direction is to directly establish the relationship between the microstructure characteristics of the alloys and superelastic properties.

## 4. Conclusions

This paper established a predictive model for superelastic strains of Ni-free superelastic titanium alloys based on machine learning methods. Furthermore, it designed new alloys through experimental validation utilizing Bayesian intelligent optimization algorithms. The main conclusions are as follows:

1. Machine learning models were established based on 125 data points. Among them, the CatBoost model constructed with features strain, T1, t1, E, MP3 and PE4 performed the best with an R^2^ of 0.83 and MAE of 0.44.
2. The features included in the final feature set, such as strain, T1, and t1 were mainly related to the processing and preparation of the alloy. The reason higher cold rolling deformation and annealing temperature were advantageous for the superelastic performance of the alloy was that high-temperature annealing after large-deformation cold rolling facilitates the formation of fine grains and strong texture.
3. Three alloy compositions including Ti-11Nb-26Zr-2Sn, Ti-12Nb-18Zr-2Sn and Ti-12Nb-16Zr-3Sn were designed using a Bayesian optimization algorithm which could quickly perform alloy composition optimization. After 60 iterations, it searched for alloy compositions with a superelastic strain greater than 6%.
4. Due to the lack of assessment of β stability in the model, only the Ti-12Nb-18Zr-2Sn and Ti-12Nb-16Zr-3Sn alloys exhibited a superelastic performance. The superelastic performance of both alloys matched the predictions of machine learning based on actual processes. After thermal treatment process optimization, they exhibited a recovery strain of 4.65% and 3.01%, respectively.

## Figures and Tables

**Table 1:** Initial feature set.

Classification	Description	Symbol
Processing process	Cold rolling reduction	strain
	Annealing temperature	T1
	Annealing time	t1
Empirical parameters of alloy design	Mo equivalent	Moeq
	Bo	Bo
	Md	Md
	Valence electron concentration	VEC
Phase formation parameters	Enthalpy of mixing	ΔHmix
	Entropy of mixing	ΔSmix
	Solid solution phase formation parameter	Ω
Element features	Atomic number	NA
	Atomic mass	MA
	Density	ρ
	Period	P
	Group	G
	Atomic radius	RA
	Covalent radius	RC
	Lattice constant	a
	Lattice constant	c
Physical and chemical features	Young’s modulus	E
	Melting point	MP
	Boiling point	BP
	Resistance	R
	Thermal conductivity	TC
	Thermal expansion coefficient	Cte
	Pauling electronegativity	PE
	Heat of fusion	Hf

**Table 2:** Hyperparameters for all machine learning models.

Models	Hyperparameters
SVR	C: 4.87, gamma: 2.03, epsilon: 4.38 × 10^−4^
CatBoost	iterations: 399, learning_rate: 0.23, l2_leaf_reg: 1.90, depth:6, subsample: 0.45, rsm: 0.95
KNN	n_neighbors: 6, weights: distance, algorithm: kd_tree
BPNN	n_layers: 2, n_units_l0: 88, n_units_l1: 40, alpha: 0.60, learning_rate_init: 0.002, momentum: 0.60, max_iter: 5000
GPR	alpha: 0.01
XGBoost	lambda: 136.32, alpha: 0.02, learning_rate: 1.49, n_estimators: 1017, max_depth: 120, gamma: 0.0003, min_child_weight: 1

**Table 3:** Nominal compositions (at.%) of new alloys.

Alloys	Nb	Zr	Sn	Ti
Ⅰ	11	26	2	61
Ⅱ	12	18	2	68
Ⅲ	12	16	3	69

**Table 4:** Chemical compositions (at.%) of the three new alloys.

Alloys	Nb	Zr	Sn	Ti
Ⅰ	10.72	26.51	2.00	60.77
Ⅱ	12.28	17.14	2.02	68.55
Ⅱ	11.62	15.89	3.12	69.37

## Data Availability

The raw data supporting the conclusions of this article will be made available by the authors on request.
